# Effects of direct-to-consumer alcohol home delivery policies on alcohol-related online searches in the United States from 2019 to 2023: A Google Trends study

**DOI:** 10.1016/j.pmedr.2025.103005

**Published:** 2025-02-15

**Authors:** McKenna Roudebush, Melissa J. Cox, Kurt M. Ribisl, Jimikaye B. Courtney

**Affiliations:** a Department of Health Behavior, Gillings School of Global Public Health, University of North Carolina at Chapel Hill, 105 Dauer Dr, Chapel Hill 27599, NC, USA; b Lineberger Comprehensive Cancer Center, University of North Carolina at Chapel Hill, 450 West Dr, Chapel Hill 27599, NC, USA; c Department of Exercise and Sports Science, College of Arts and Sciences, University of North Carolina at Chapel Hill, 209 Fetzer Hall, Chapel Hill 27599, NC, USA

**Keywords:** Alcohol use, Internet, Public health policy, Alcohol-related harms, COVID-19 pandemic

## Abstract

**Objective:**

This study examined the effects of direct-to-consumer alcohol home delivery (DTC) policies enacted in response to the COVID-19 pandemic on online searches for alcohol delivery and alcohol-related harms in the United States.

**Methods:**

Google Trends data (2019–2023) were used to assess weekly relative search volume (RSV) for three terms reflecting 1) DTC and 2) potential harms associated with expanded DTC: *alcohol delivery*, *alcohol poisoning*, and *alcoholic*. RSV for each term was extracted for states representing four DTC policy contexts (no DTC [South Carolina], no change in existing DTC policy [Pennsylvania], new DTC policy [Georgia], expanded DTC policy [California]). Interrupted times series analyses evaluated the effect of time and policy implementation on RSV in states introducing or expanding DTC policies, while linear regressions evaluated the effect of time where DTC policies did not change.

**Results:**

There was a significant positive effect of time on RSV for *alcohol delivery* in the state that introduced a new DTC policy (β = 0.1, *p* = 0.02), with RSV declining over time following policy implementation (β = −0.1, *p* = 0.001). In the state that expanded DTC policy, *alcohol delivery* RSV increased immediately after policy expansion (β = 20.9, *p* < 0.001) and declined in subsequent months (β = −1.5, *p* < 0.001). RSV for alcohol-related harms fluctuated across states.

**Conclusions:**

Online search interest related to alcohol delivery and harms varied post-DTC policy implementation. These findings highlight the potential of search trends to provide real-time insights into public health impacts of evolving alcohol policies.

## Highlights

• Direct-to-consumer alcohol home delivery policies expanded post-COVID-19 pandemic.
• Alcohol delivery searches rose post-policy change, then declined in some states.
• Alcohol-related harms searches fell after policy changes in certain states.

## 1 Introduction

Excessive alcohol use is a significant public health issue in the United States (US) with consequences ranging from acute harms (e.g., alcohol poisoning) to chronic conditions (e.g., alcohol use disorder) (Hingson et al., 2017). Alcohol-attributable mortality has increased in the US in the past 20 years (Maleki et al., 2023), particularly from 2016 to 2021, when approximately 488 people died per day from excessive alcohol use (Esser et al., 2024). The rise in alcohol-attributable deaths aligns with the onset of the COVID-19 pandemic (referred to as “the pandemic”) in March 2020, when alcohol consumption patterns rapidly shifted in the US. One study of US adults age ≥ 21 found a 21 % increase in binge drinking from February to April 2020 (Barbosa et al., 2021). Prevalence of daily alcohol consumption peaked among adults ages 35–50 in 2020 (12.0 %; Patrick et al., 2024). Simultaneously, alcohol sales surged during the pandemic (Hu et al., 2021; Slater and Alpert, 2024). From March to September 2020, retail alcohol sales increased by 20 % compared to the previous year (Castaldelli-Maia et al., 2021).

During the early pandemic, there were also changes to alcohol control policies that increased alcohol availability. When businesses were facing economic challenges due to COVID-19 control measures enacted at the onset of the pandemic, many states introduced or increased permissions for direct-to-consumer (DTC) alcohol home delivery, which allows consumers to purchase alcohol for delivery to their home (Colbert et al., 2021; National Institute on Alcohol Abuse and Alcoholism [NIAAA], 2022; Trangenstein et al., 2023a). In this paper, the term “DTC alcohol home delivery” encapsulates any instance in which alcohol is purchased and delivered to the consumer's home, including online purchases of alcohol from national retailers shipped across state lines and purchases from local retailers for same-day delivery by the business or a third-party delivery app. While the rapid expansion of DTC alcohol home delivery was initially framed as temporary pandemic relief, many states have since made provisions permanent (Lemp et al., 2024).

Permissive DTC delivery policies increase alcohol availability by expanding how alcohol may be obtained and the contexts in which it may be consumed (Lemp et al., 2024). Increased alcohol availability is associated with excessive alcohol use and elevated risk of experiencing alcohol-related harms (Fitterer et al., 2015; Gruenewald, 2011). While the impact of expanded DTC alcohol delivery on alcohol-related harms is an emerging topic, a recent study demonstrated that using DTC alcohol delivery was associated with elevated alcohol consumption and increased negative consequences (Courtney et al., 2025). One study on alcohol purchasing behaviors during the pandemic found that US adults who purchased alcohol via DTC delivery consumed alcohol on more days per month, consumed more drinks per drinking occasion, and were more likely to engage in binge drinking than those who purchased alcohol by other methods (Grossman et al., 2022). From December 2019 to December 2020, DTC use was also associated with purchasing alcohol in greater quantities compared to purchasing alcohol in-store (Trangenstein et al., 2023b). Furthermore, previous studies have demonstrated that DTC alcohol delivery may increase alcohol access among underage individuals due to poor adherence to age verification procedures (Noel and Rosenthal, 2023; Williams and Ribisl, 2012), which is concerning given that underage drinking contributes to a range of negative consequences (Harding et al., 2016; Marshall, 2014). Other research has examined how DTC use and alcohol-related harms differ across states with varying DTC policy contexts (Courtney et al., 2025). Public health recommendations suggest limiting alcohol availability as an evidence-based way to reduce excessive alcohol consumption and related harms; therefore, research on the effects of expanded DTC state policies is warranted (World Health Organization, 2019).

The rapid growth of DTC delivery since the onset of the pandemic necessitates novel strategies for examining its impacts. Given that Internet searches are used by many consumers to both identify DTC sellers and pursue health-related information, Google Trends is a useful tool for examining population-level interest in DTC alcohol sales and related harms. Google Trends is an open access online repository of Google Search data that enables users to examine geographical and time-based trends in searches for specified terms (Nuti et al., 2014). Public health research has utilized Google Trends for a variety of purposes, ranging from monitoring disease outbreaks such as COVID-19 to gauging public engagement with health-related behaviors such as substance use (Arora et al., 2019; Ayers et al., 2014; Mavragani et al., 2018; Nuti et al., 2014). Google Trends has also been used to assess the impact of alcohol policy changes on online searches related to alcohol use behaviors and related health consequences in India (Ghosh et al., 2021; Singh et al., 2021). However, to our knowledge, no study has examined online searches for DTC alcohol delivery and alcohol-related harms in the US since the recent proliferation of DTC alcohol policies. Therefore, this study examined online search trends related to DTC alcohol delivery and alcohol-related harms from pre-pandemic (January 1, 2019) to late-pandemic (October 31, 2023) across states with different DTC alcohol delivery policy contexts.

## 2 Methods

### 2.1 Search term development

Search terms were selected via a multi-step process. First the research team collaboratively identified initial search terms related to three broad themes of interest: alcohol access (order alcohol online, alcohol delivery), acute alcohol-related harms (alcohol withdrawal, blackout drunk, and alcohol poisoning), and long-term alcohol-related harms (alcoholism, alcoholic, alcohol dependence, and alcohol addiction). The term alcoholic is not used by the Centers for Disease Control and Prevention or the field because it can be stigmatizing but it was selected for this study because it is a commonly used term in Google searches.

Second, each search term was tested in Google Trends to identify its top related searches within the period of interest (1/1/2019–10/31/2023) across the US. Google Trends allows users to specify a search topic category to minimize unrelated results. Searches related to alcohol access were conducted within the “Alcoholic Beverages” category, while searches related to alcohol-related harms were conducted within the “Health” category. We used search term operators to remove searches related to pop culture, news events and other unrelated categories (e.g., “-non” was included as a search operator for the *alcoholic* term to remove searches for nonalcoholic beverages). This testing yielded five additional terms related to online alcohol access (*buy liquor online, order liquor online, liquor delivery, wine delivery, beer delivery*); however, these search terms were excluded to limit the search to terms with broad applicability across all alcohol types. No new relevant searches were found for acute or long-term alcohol harms.

Given that Google Trends does not allow users to download aggregated data for multiple terms, and previous research has documented data fluctuations for a single term according to the date and time of data extraction (Tran et al., 2017), the research team decided to assess one search term per theme for parsimony. Terms were compared in Google Trends to identify the most popular search term within each theme. In the online alcohol access theme, *alcohol delivery* was the most popular search term. *Alcohol poisoning* was most frequently searched in the acute harms theme, and *alcoholic* was the most popular search term in the long-term harms theme. The full search strategy is available in Table 1.

**Table 1:** Google Trends Search Strategy.

	*alcohol delivery*	*alcohol poisoning*^1^, ^2^	*alcoholic*^1^, ^2^, ^3^
*Search Variables*			
Access Date	12/4/2023–12/10/2023	12/4/2023–12/10/2023	12/4/2023–12/10/2023
Time Period	1/1/2019–10/31/2023	1/1/2019–10/31/2023	1/1/2019–10/31/2023
Query Category	Alcoholic beverages	Health	Health
*Search Input*			
Geographic Regions	Georgia, California, Pennsylvania, South Carolina	Georgia, California, Pennsylvania, South Carolina	Georgia, California, Pennsylvania, South Carolina
Search Operators	None	-tiktok; −euphoria; −dogs	-keto; −non; −nancy; −love; −olive
Full Search Input	“alcohol delivery”	alcohol poisoning -tiktok -euphoria -dogs	alcoholic -keto -non -nancy -love -olive

*Notes.* This checklist was developed in accordance with best practices for Google Trends search strategy documentation recommended by Nuti et al. (2014).

1 Media or cultural events may bias search results, necessitating the use of search operators to remove unrelated results. For alcohol poisoning, the minus symbol indicates that searches which include “alcohol poisoning” and return results related to the social media app TikTok, the television show Euphoria, or dogs should not be included. For the term alcoholic, the minus symbol indicates that searches which include “alcoholic” and return results related to the keto diet, the word “non” (e.g., nonalcoholic), the name Nancy (e.g., Nancy Reagan), the word “love,” or the word “olive” should not be included.

2 Quotations were not used in the full search input because Google Trends does not allow quotations to be used alongside search operators.

3 The term *alcoholic* is not used by the Centers for Disease Control and Prevention or in the field because it can be stigmatizing. It was included in this study because it is a term commonly used in Google searches.

### 2.2 Data extraction

Google Trends provides a search term's normalized relative search volume (RSV) for a specific region and period. RSV indicates how frequently a term is searched compared to the total number of searches conducted in the defined region and period. Data are normalized to the highest search proportion, which corrects for changes in absolute search volume. RSV ranges from zero to 100, where 100 represents the peak search volume. In December 2023, we downloaded weekly RSV data for each term from January 1, 2019, through October 31, 2023. We conducted one download for each search term every day over seven days and calculated a composite average weekly RSV based on those downloads to account for fluctuation in reported RSV (Tran et al., 2017). This study utilized public, anonymized data and was therefore exempt from ethical compliance.

### 2.3 State alcohol policy context

Prior to data collection, an alcohol policy advisory board comprised of experts from government, private, and academic institutions met to determine state policy environments based on DTC delivery policies enacted pre- and post-pandemic. Alcohol policy data were extracted from the Alcohol Policy Information System detailing updates to DTC policies for on- and off-premises establishments (NIAAA, 2022). This yielded four broad descriptors to define state-level DTC policy contexts: 1) *No Delivery* (South Carolina) pertains to states which never allowed DTC alcohol delivery and did not change that policy during or post-pandemic; 2) *No Change in Delivery* (Pennsylvania) includes states that allowed DTC pre-pandemic and did not expand their DTC policy; 3) *Expanded Delivery* (California) refers to states which did allow DTC alcohol delivery pre-pandemic and expanded their DTC policy after the onset of the pandemic. In California, the expansion allowed for delivery of mixed drinks in addition to beer and wine; and 4) *New Delivery* (Georgia) captures states which did not allow DTC pre-pandemic and introduced DTC alcohol policies during the pandemic. In this case, no delivery of alcohol was previously allowed while legislation passed to allow on-premise beer and wine delivery as well as off-premise delivery for any liquor type. Data were extracted for the most populous state in each policy context since Google Trends does not allow multiple geographic regions to be selected in one search. Additional details regarding the policy context development process can be found in Courtney et al. (2025). Key policies and dates for the state representing each policy context are listed in Table 2.

**Table 2:** Summary of Key Direct-to-Consumer Alcohol Home Delivery Policies and Dates in the United States, 2019—2023.

*Policy Context*	*State*	*Pre-Pandemic Policy*	*Pandemic Policy*	*Final Policy Situation*	*Date of Direct-to-Consumer Policy Implementation*^1^	*Date of First Stay-at-Home Order*^2^	*Date Stay-at-Home Order Lifted*^2^
New Delivery^3^	Georgia	No on- or off-premise delivery allowed	On-premise beer and wine delivery allowed, off-premise delivery allowed for any liquor type	On-premise beer and wine delivery allowed, off-premise delivery allowed for any liquor type	8/3/2020	4/3/2020	4/30/2020
Expanded Delivery^4^	California	On-premise beer and wine delivery allowed, off-premise delivery allowed	On- and off-premise delivery of any liquor type allowed	On-premise beer and wine delivery allowed, off-premise delivery allowed for any liquor type	3/19/2020	3/19/2020	6/15/2021
No Change in Delivery^5^	Pennsylvania	Both on- and off-premise delivery allowed, wine and beer only	Both on- and off-premise delivery allowed, wine and beer only	Both on- and off-premise delivery allowed, wine and beer only	-	4/1/2020	4/30/2020
No Delivery^6^	South Carolina	No on- or off-premise delivery allowed	No on- or off-premise delivery allowed	No on- or off-premise delivery allowed	-	4/7/2020	5/12/2020

1 National Institute on Alcohol Abuse and Alcoholism (NIAAA), 2022.

2 Centers for Disease Control and Prevention (CDC), 2021.

3 New Delivery: Did not allow delivery prior to the pandemic, with on- and/or off-premise delivery allowed after pandemic onset.

4 Expanded Delivery: Allowed on- and/or off-premise delivery prior to pandemic with expanded permissions after pandemic onset.

5 No Change in Delivery: Allowed on- and/or off-premise delivery prior to pandemic with no changes after pandemic onset.

6 No Delivery: did not allow on- or off-premise delivery prior to or after pandemic onset.

### 2.4 Predictors

Time was assessed via weekly RSV for each search term downloaded from January 1, 2019, through October 31, 2023. This resulted in 252 total weekly time points, represented by a continuous variable 1–252.

The date of DTC policy implementation in states representing the New Delivery and Expanded Delivery policy contexts was identified using the Alcohol Policy Information System (NIAAA, 2022). A dichotomous policy shock variable was created to represent the immediate effect of DTC policy implementation (0 = pre-DTC policy implementation; 1 = post-DTC policy implementation). A continuous policy trend variable was created to represent the effect of DTC policy implementation over time, beginning with 1 at the week in which DTC policy went into effect. For example, the date of DTC policy implementation in the state representing the New Delivery policy context (Georgia) was August 3, 2020, which aligned with the RSV download week August 8, 2020; therefore, the continuous policy trend variable was 0 pre-August 8, 2020 and began at 1 post-August 8, 2020.

### 2.5 Statistical analyses

**Fig. 1:**
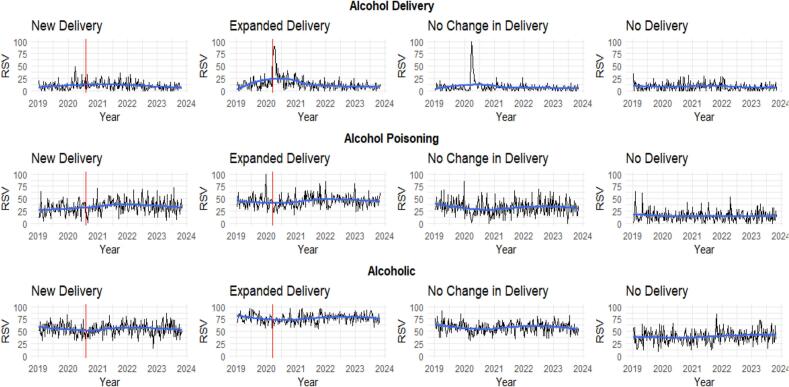
Online Search Trends for Selected Terms by Policy Context in the United States^1,2^, 2019—2023. Notes. RSV = relative search volume. The red line indicates the date of policy implementation. ^1^State policy contexts are: New Delivery (Georgia): Did not allow delivery prior to the pandemic, with on- and/or off-premise delivery allowed after pandemic onset; Expanded Delivery (California): Allowed on- and/or off-premise delivery prior to pandemic with expanded permissions after pandemic onset; No Change in Delivery (Pennsylvania): Allowed on- and/or off-premise delivery prior to pandemic with no changes after pandemic onset; No Delivery (South Carolina): Did not allow on- or off-premise delivery prior to or after pandemic onset. ^2^The RSV values do not reach 100 % due to creating an average RSV per week.

Interrupted time series (ITS) analyses were conducted via a linear regression model to examine effects of time and DTC policy implementation on online searches for selected terms in states that introduced or expanded their DTC alcohol policy. ITS methods were not applied to states that did not alter their DTC policy during the study period, meaning there was no policy to interrupt the time series; a linear regression model was used to assess the effect of time on online searches in these states. All models were first tested for linear and non-linear effects of time and modeled to include up to the highest order of time that was significant, after which DTC policy implementation shock and trend predictors were added to the models. Autocorrelation was assessed via the Durbin-Watson test (Durbin and Watson, 1971). A lag term was added to correct models where autocorrelation was present (Wilkins, 2018). Graphs of online search trends across the examined period showed *alcohol delivery* searches peaking in late March 2020 across several policy groups (see Fig. 1). Consequently, we hypothesized that the onset of the pandemic may have influenced search trends prior to expanded DTC policy implementation. We conducted sensitivity analyses to examine the pandemic effect. First, a dichotomous pandemic shock variable was created where the onset of the pandemic was defined as the date of the first stay-at-home order in each state (0 = pre-pandemic; 1 = post-pandemic). A continuous policy trend variable was then constructed to capture the effect of the pandemic over time, beginning with 1 at the week in which the first stay-at-home order went into effect in that state. The date of DTC policy implementation coincided with the first stay-at-home order in the Expanded Delivery group; therefore, additional analyses were not conducted for this policy group. The model results with the added pandemic variables aligned with the original policy only models; therefore, the original models were retained in final analyses. All models were fitted in R version 4.2.2 (R Core Team, 2020).

## 3 Results

### 3.1 Alcohol delivery

In the state representing the New Delivery policy context (Georgia), there was a significant overall positive effect of time on RSV for *alcohol delivery* (β = 0.1, *p* = 0.02) (Table 3). There was no significant change in RSV for *alcohol delivery* immediately following the implementation of a new DTC delivery policy (β = 0.3, *p* = 0.9), though RSV declined from the date of DTC policy implementation through the remainder of the study period (β = −0.1, *p* = 0.001). In the state representing the Expanded Delivery policy context (California), RSV for *alcohol delivery* increased immediately following DTC policy expansion (β = 20.9, *p* < 0.001), but declined over time (β = −1.5, *p* < 0.001). There were no significant effects of time on RSV for *alcohol delivery* in either the states representing the No Change in Delivery (Pennsylvania) or No Delivery (South Carolina) policy contexts.

**Table 3:** Effects of Time and Direct-to-Consumer Alcohol Home Delivery Policy Implementation on Online Searches for Selected Terms in the United States, 2019—2023.

	New Delivery^1^, ^4^			Expanded Delivery^1^, ^5^			No Change in Delivery^1^, ^6^			No Delivery^7^		
*Predictors*	*Estimate*	*Standard Error*	*p*^2^	*Estimate*	*Standard Error*	*p*^2^	*Estimate*	*Standard Error*	*p*^3^	*Estimate*	*Standard Error*	*p*^3^
*Alcohol Delivery*												
Intercept	6.4	1.7	<0.001**	12.7	4.3	0.003*	2.6	3.1	0.4	9.1	0.9	<0.001**
Time	0.1	0.0	0.02*	−0.5	0.3	0.1	−0.1	0.2	0.8	−0.0	0.0	0.2
Quadratic Time				0.0	0.0	0.1	0.0	0.0	0.6			
Cubic Time				0.0	0.0	0.7	0.0	0.0	0.5			
Policy (Shock)	0.3	2.1	0.9	20.9	4.2	<0.001**						
Policy (Trend)	−0.1	0.0	0.001*	−1.5	0.3	<0.001**						
*Alcohol Poisoning*												
Intercept	23.1	4.8	<0.001**	42.7	4.5	<0.001**	39.5	4.6	<0.001**	15.6	1.3	<0.001**
Time	0.3	0.3	0.3	0.0	0.4	0.9	−0.2	0.3	0.5	−0.0	0.0	0.4
Quadratic Time	0	0.0	0.9	0.0	0.0	0.8	0.0	0.0	0.8			
Cubic Time	0	0.0	0.4	0.0	0.0	0.4	0.0	0.0	0.5			
Policy (Shock)	−4.4	5.1	0.4	−13.8	4.3	0.001*						
Policy (Trend)	0.6	0.3	0.04*	0.4	0.4	0.3						
*Alcoholic*												
Intercept	52.8	4.9	<0.001**	63.5	7.4	<0.001**	63.6	3.4	<0.001**	36.0	1.6	<0.001**
Time	0.2	0.4	0.6	−0.0	0.9	0.9	−0.2	0.2	0.3	0.03	0.0	0.02*
Quadratic Time	0	0.0	0.8	0.0	0.1	0.7	0.0	0.0	0.8			
Cubic Time	0	0.0	0.4	0.0	0.0	0.5	0.0	0.0	0.7			
Policy (Shock)	4.4	4.7	0.4	4.6	3.9	0.2						
Policy (Trend)	1.3	0.4	0.003*	0.7	0.5	0.2						

*Notes.* *p < 0.01; **p < 0.001. Models were calculated independently and presented together to illustrate trends across policy contexts.

1 Nonlinear effects of time higher than the cubic order were found for some terms in the New Delivery, Expanded Delivery, and No Change policy contexts.

2 Interrupted time series analyses using a linear regression model.

3 Linear regression models.

4 New Delivery (Georgia): Did not allow delivery prior to the pandemic, with on- and/or off-premise delivery allowed after pandemic onset.

5 Expanded Delivery (California): Allowed on- and/or off-premise delivery prior to pandemic with expanded permissions after pandemic onset.

6 No Change in Delivery (Pennsylvania): Allowed on- and/or off-premise delivery prior to pandemic with no changes after pandemic onset.

7 No Delivery (South Carolina): did not allow on- or off-premise delivery prior to or after pandemic onset.

### 3.2 Alcohol poisoning

In the New Delivery policy context (Georgia), there was no significant change in RSV for *alcohol poisoning* immediately following the implementation of a new DTC delivery policy (β = −4.4, *p* = 0.4), though RSV significantly increased from the date of DTC policy implementation through the remainder of the study period (β = 0.61, *p* = 0.038). In the Expanded Delivery policy context (California), there was a sharp significant decrease in RSV for *alcohol poisoning* immediately following the implementation of DTC policy expansion (β = −13.8, *p* = 0.001). There was no significant effect of time on RSV for *alcohol poisoning* searches over the study period in the No Change in Delivery (Pennsylvania) and No Delivery (South Carolina) policy contexts.

### 3.3 Alcoholic

In the New Delivery policy context (Georgia), there was no significant change in RSV for *alcoholic* immediately following the introduction of a new DTC delivery policy (β = 4.4, *p* = 0.4), but the RSV significantly increased from the date of DTC policy implementation throughout the remainder of the study period (β = 1.3, *p* = 0.003). In the No Delivery policy context (South Carolina), there was a significant and positive effect of time on RSV for the term *alcoholic* (β = 0.03, *p* = 0.02). No significant associations with RSV for *alcoholic* were detected in the states representing Expanded Delivery (California) or No Change in Delivery (Pennsylvania) policy contexts.

## 4 Discussion

Our findings suggest that Google searches for alcohol delivery and alcohol-related harms fluctuated from pre- to late-pandemic across states with varying DTC alcohol delivery policy contexts. Online searches for *alcohol delivery* generally declined from the time of policy introduction or expansion through late pandemic in the New and Expanded Delivery policy contexts. Search interest in alcohol-related harms terms (*alcohol poisoning* and *alcoholic*) varied across policy contexts. These findings can contribute to the growing literature examining the effects of expanding DTC alcohol delivery policies on alcohol access and related consequences.

Expanded DTC alcohol delivery policies transformed alcohol access in the US following the pandemic onset. The number of people living in states that permitted DTC alcohol sales increased by 284 % compared to levels before March 2020 (Trangenstein et al., 2023b). We found that searches for *alcohol delivery* spiked immediately following early pandemic DTC expansion in the Expanded Delivery context, which could be due to residents of this state being familiar with how to purchase alcohol via DTC methods. Similarly, we observed that the other context that permitted DTC alcohol delivery prior to the pandemic (No Change in Delivery [Pennsylvania]) showed a pronounced increase in *alcohol delivery* searches during the early pandemic period (Fig. 1). In contrast, there was no immediate effect of introducing DTC alcohol delivery policy on *alcohol delivery* searches in New Delivery context. This may be attributed to the timing of policy implementation as DTC delivery was first permitted in early August 2020 after lockdowns had eased in this state (Georgia) (Centers for Disease Control and Prevention, 2021). Prior work has demonstrated that DTC delivery was frequently used to obtain alcohol during early pandemic lockdowns (e.g., March–May 2020) (Grossman et al., 2020, Grossman et al., 2022; Trangenstein et al., 2023a); August 2020 may have been too late to have an immediate impact on *alcohol delivery* searches. Indeed, prior research found that DTC use declined as pandemic restrictions were lifted (Grossman et al., 2020, Grossman et al., 2022; Trangenstein et al., 2023a). We found a similar pattern of declining *alcohol delivery* searches in both states representing the New and Expanded Delivery contexts from policy implementation through October 2023. These findings suggest that general awareness of new DTC alcohol home delivery policies may require time to diffuse. Given that policies to regulate alcohol availability continue to fluctuate post-pandemic, Google Trends is a helpful tool to quickly identify trends in search interest for related topics (e.g., *alcohol delivery*). Such timely assessment can help to inform and monitor the public health response to policy change.

This study also found a significant decline in searches for *alcohol poisoning* immediately following policy implementation in the Expanded Delivery context, with no significant change over the remainder of the study period. This may be due in part to the fact that California, the state representing the Expanded Delivery policy context, expanded its DTC alcohol policy mid-March 2020, when COVID-19 concerns likely shifted focus away from other health issues and contributed to an avoidance of medical care (Czeisler, 2020). Conversely, Google searches for *alcoholic* and *alcohol poisoning* increased in the New Delivery context from policy implementation through October 2023. While existing research leaves unanswered questions regarding the effect of the pandemic on alcohol-related harms, early studies found an increase in some alcohol-related problems reported during the pandemic (Pollard et al., 2020; Wardell et al., 2020), including alcohol-related emergency department visits (Esser et al., 2022). Initial research suggests that adults living in a state with expanded DTC alcohol delivery policies had higher odds of using alcohol delivery, which in turn was associated with consuming more drinks per week, engaging in binge drinking on more days per month, and experiencing greater negative consequences of drinking (Courtney et al., 2025; Grossman et al., 2022). The uptick in interest in these topics (*alcohol poisoning* and *alcoholic*) coupled with preliminary findings regarding DTC use and alcohol-related harms warrants further inquiry to examine the relationship between search interest and experience of actual harm. For example, previous research on help-seeking online searches for substance use treatment found that search interest predicted demand for substance use treatment and related emergency department visits (Patton et al., 2022). While longitudinal research monitoring the longer-term effects of DTC alcohol policies on alcohol-related harms is needed (Courtney et al., 2025), these findings provide a real-time snapshot of search interest in topics reflective of alcohol-related harms and may be a useful early warning signal of the impact of policy change on substance use behaviors that are central to prevention and treatment efforts.

### 4.1 Limitations

This study utilizes an innovative data source to examine online search trends in the context of a fluctuating policy environment; however, several key limitations exist. While Google Trends offers valuable insights into public interest in topics over time, the data represent the relative search volume rather than absolute search volume. Though this helps to normalize the data for comparisons across groups, this may obscure the magnitude of search activity for a specific term (Alibudbud, 2023). Previous studies have found that RSV fluctuates according to when it is downloaded (Tran et al., 2017), though we have addressed this according to current best practices by performing repeated data extractions and calculating an average RSV for each time point. While we selected the most popular search terms according to available data to maintain parsimony, other terms may have also been suited for inclusion in this study. Further research may utilize novel methods of selecting search terms, such as surveying priority populations (Cho et al., 2013), to ensure the most salient terms are included. Additionally, cultural events may bias available Google Trends data, resulting in unrelated searches contributing to a term's RSV. Specifying operators and search criteria mitigate this risk, though it is possible some unrelated searches may not have been filtered out by the specifications we set. We assessed online search trends in the most populous state from each policy group to minimize the total number of models calculated given that Google Trends does not allow for multiple geographic regions to be selected in a single search. As such, our findings may not be applicable to other states with the same DTC alcohol delivery policy context. Finally, previous research has demonstrated a rise in app-based delivery platforms following the onset of the pandemic (Duthie et al., 2023). The documented decline in *alcohol delivery* searches may be due in part to in-app searches not captured by Google Trends. Future research may utilize in-app purchase data to further examine alcohol delivery trends.

## 5 Conclusion

This study examined trends in Google searches related to alcohol delivery and alcohol-related harms in four US states representing distinct DTC policy contexts from pre- to late-pandemic. Findings suggest that online searches for alcohol delivery increased immediately following DTC policy expansion in states with pre-existing permissive DTC delivery environments and then steadily decreased over time as pandemic lockdowns lifted and on-premises alcohol outlets re-opened. Trends for alcohol-related harms search terms varied by DTC policy context. Overall, these results indicate that online interest in alcohol delivery and alcohol-related harms fluctuated over the course of the pandemic across states with varying DTC policy contexts. Insights from this research can contribute to current knowledge of the public health implications of DTC alcohol delivery at the state level and inform efforts to reduce alcohol-related harms.

## Disclosure of Funding

This study was supported by the 10.13039/100000030Centers for Disease Control and Prevention of the U.S. Department of Health and Human Services (HHS) as part of a financial assistance award with 100 % funded by CDC/HHS (Cooperative Agreement Number U48 DP006400). The findings and conclusions in this report are those of the author(s) and do not necessarily represent the official position of the CDC or HHS.

## CRediT authorship contribution statement

**McKenna Roudebush:** Writing – original draft, Project administration, Formal analysis, Data curation. **Melissa J. Cox:** Writing – review & editing, Methodology, Conceptualization. **Kurt M. Ribisl:** Writing – review & editing, Funding acquisition, Conceptualization. **Jimikaye B. Courtney:** Writing – review & editing, Supervision, Project administration, Methodology, Conceptualization.

## Declaration of competing interest

The authors declare that they have no known competing financial interests or personal relationships that could have appeared to influence the work reported in this paper.

## Data Availability

Data will be made available on request.
